# Influence of ethnicity on plantar loading of the paediatric foot: an exploratory analysis

**DOI:** 10.1186/1757-1146-8-S2-O28

**Published:** 2015-09-22

**Authors:** Stewart C Morrison, Stephen Cousins, Charlotte Vannet, Wendy Drechsler

**Affiliations:** 1 Human Motor Performance Group, School of Health, Sport and Bioscience, University of East London, E15 4LZ, UK; 2 Faculty of Health Sciences, La Trobe University, Victoria, 3350, Australia

**Keywords:** ethnic group, foot, paediatrics

## Background

Variance in the morphological characteristics and frequency of pathology in the paediatric foot are thought to exist between ethnic groups. Differences in characteristics have primarily been studied from a footwear perspective with limited research describing the effects of ethnicity on dynamic foot loading. The aim of this study was to investigate the influence of ethnicity on loading characteristics of the paediatric foot.

## Methods

Dynamic plantar pressures were measured in 93 children aged 7-11 years (mean age 9.4 +1.6 years) using the MatScan^®^ 3150 pressure platform. Participants were recruited from local schools in East London and stratified into four ethnic groups (white, mixed, black and asian). Mean peak plantar pressure at six different segments across the plantar foot was calculated from three walking trials using the midgait protocol. Multiple linear regression was used to test for ethnic effects, whilst controlling for gender and body mass.

## Results

Significant differences for mean pressure at the lateral heel (*p* = ? 0.05) and the medial heel (*p* = ? 0.05) were identified. These were principally driven by differences between the mixed ethnic group for loading at the medial heel (*p* = ? 0.05), which showed significantly lower mean peak pressure than the white ethnic group. Significantly lower peak pressures were also identified at the lateral heel for both the mixed (*p* = ? 0.05) and black (*p* = ? 0.05) ethnic groups.

## Conclusion

Significant differences in peak plantar pressure across the heel were identified in an ethnically diverse sample of children aged 7-11 years from East London. These findings suggest that differences in foot loading characteristics exist between the groups and may be explained by variances in foot position during gait. It is anticipated that this research will increase understanding of typical foot development in different ethnic groups.

